# Microsatellite Markers of Willow Species and Characterization of 11 Polymorphic Microsatellites for *Salix eriocephala* (Salicaceae), a Potential Native Species for Biomass Production in Canada

**DOI:** 10.3390/plants2020203

**Published:** 2013-03-27

**Authors:** Aurélien Lauron-Moreau, Frédéric E. Pitre, Luc Brouillet, Michel Labrecque

**Affiliations:** Institut de recherche en biologie végétale, Université de Montréal, 4101 Sherbrooke Est, Montréal, QC H1X 2B2, Canada; E-Mails: aurelien.lauron-moreau@umontreal.ca (A.L.-M.); frederic.pitre@umontreal.ca (F.E.P.); luc.brouillet@umontreal.ca (L.B.)

**Keywords:** allele data, microsatellite markers, Salicaceae, *Salix eriocephala*, willow

## Abstract

Biomass produced from dedicated plantations constitutes a source of renewable energy and is expected to play an important role in several countries in the coming decades. The cultivation of woody crops such as willows therefore raises several environmental issues. In North America, several native willows are potentially interesting for biomass producers. Willow trees are diverse but few species used for environmental applications have been the object of molecular genetic studies. Based on the sequenced poplar genome, 24 microsatellite markers were assayed on five native North American willow species: *Salix amygdaloides*, *S. discolor*, *S. eriocephala*, *S. interior* and *S. nigra*. Polymorphic microsatellite markers were used to characterize the allele data on the shrub *Salix eriocephala*, a North American species with economic potential. Eleven markers amplified and confirmed the potential of this species. Analysis of samples from six populations in eastern Canada showed that all markers were variable as well as polymorphic in at least one population. The number of alleles per locus ranged from 1 to 9 (mean 2.95) and showed that these microsatellite markers can be used to assess genetic diversity of North American willow species.

## 1. Introduction

Genus *Salix* L. (Salicaceae) includes about 500 species worldwide, concentrated in temperate and cold regions of the Northern Hemisphere. Although it presents greater diversity in areas such as China (around 275 species; [1]), more than 100 species are found in North America [2]. Willow species are cultivated worldwide for the production of biomass and for environmental applications [3] using short-rotation intensive culture (SRIC). Some willow species and cultivars have been favored due to their high biomass yield [4,5]. In North America, most of the cultivars used for biomass production are of exotic origins. The use of native species could represent several advantages to stimulate the deployment of bioenergy plantations. In addition to their higher level of social acceptability, it is believed that native species will be better adapted to the pedoclimatic conditions characterizing North American regions. They are also characterized by a higher genetic diversity within their geographical distribution than introduced cultivars and they could be exploited for diverse applications [6]. 

The North American willow species, *Salix amygdaloides*, *S. discolor*, *S. eriocephala*, *S. interior* and *S. nigra*, have morphological traits that suggest high biomass potential. These shrubs can grow rapidly to 6–8 meters and are widespread in eastern North America [2]. Among these, *Salix eriocephala* is of particular interest because of its extensive North-South distribution and adaptation to a range of climatic conditions. Knowledge of the genetic diversity of populations is a prerequisite to breeding programs aimed at improving biomass production. At a broader scale, given that half the current range of this species was once covered by Quaternary glaciers, genetic information may provide clues to the species population structure following recolonization of glaciated areas [7]. Barker *et al*. [8] developed 46 microsatellite markers in *Salix*, of which 17 were polymorphic (2–22 alleles per locus). Two studies derived microsatellite markers for *Salix* from the related *Populus trichocarpa* (Salicaceae) genome [9] to evaluate genetic diversity: Puschenreiter *et al*. [10] successfully amplified 73% of the *Populus* SSRs assayed in *S. caprea* (only 10% were polymorphic), each of which was functional on a variable number of species among the four species assayed; Lin *et al*. [6] used eight microsatellite markers, from both *Populus* and the Barker study (PMGC2709, PMGC2889, Karp_SB24, Karp_SB199, Karp_SB493, Karp_W293, Karp_W504 and Karp_W784) to compare the genetic diversity of *S. eriocephala* to that of *S. purpurea* (introduced willow) at a regional level.

Here, we assayed 24 microsatellite markers on five willow species and characterized 11 polymorphic microsatellite markers for *S. eriocephala* in widely distributed populations in eastern Canada. Our objectives were: (1) to determine whether these markers were present in the North American *Salix* species studied, and (2) to evaluate polymorphisms within the species *S. eriocephala*.

## 2. Results and Discussion

In total, twelve microsatellites were amplified with *Salix* species of this study and five markers were shared in these willows (Table 1). In addition, WPMS_7 amplified in *S. amygdaloides*, *S. discolor*, *S. interior* and *S. nigra*; ORPM_207 and PMGC_2658 in *S. amygdaloides*, *S. eriocephala* and *S. nigra*; PMGC_2315 in *S. amygdaloides*, *S. eriocephala* and *S. interior*; and WPMS_18 in *S. discolor* and *S. eriocephala*. Two markers (ORPM_21 and ORPM_349) were present only in *S. eriocephala*.

**Table 1:** Characteristics of 24 microsatellite markers used for five willow species, of which 11 successfully amplified for *Salix eriocephala*. Information on each primer pair includes locus name, expected size (bp) in poplar, forward and reverse sequences, repeat motif, successful amplification with populations of *S. amygdaloides* (Sa), *S.discolor* (Sd), *S.eriocephala* (Se), *S. interior* (Si) and *S. nigra* (Sn), and number of cycles (Ca).

Locus ^a^	Expected size (bp) in poplar	Primers sequences (5'–3')	Repeat motif	Successful amplification with populations of					Number of cycles C_a_
				Sa	Sd	Se	Si	Sn	
GCPM_1011	221	F: ATGAAATAATCGTTTGGTGC	(AT)_11_	+	+	+	+	+	27
		R: CACCCGAGTTTATCTCACTC							
GCPM_1037	123	F: ATGAAATTCGCAAAGTCAGT	(TA)_11_	+	+	+	+	+	33
		R: AAAAGAGGAAATTACGGTCC							
GCPM_1043	154	F: TTTCCATGTAGTATTACTCCTTTCT	(AT)_21_	-	-	-	-	-	33
		R: ATGCGTACCTTAGTGGAAGA							
ORPM_16	238	F: GCAGAAACCACTGCTAGATGC	(CTT)_15_	-	-	-	-	-	33
		R: GCTTTGAGGAGGTGTGAGGA							
ORPM_21	230	F: GGCTGCAGCACCAGAATAAT	(AG)_4_	-	-	+	-	-	25
		R: TGCATCCAAAATTTTCCTCTTT							
ORPM_23	197	F: ATTCCATTTGGCAATCAAGG	(AT)_6_(AG)_6_	-	-	-	-	-	33
		R: CCCTGAAAGTCACGTCTTCG							
ORPM_29	206	F: TGGTGATCCAGTTTTGGTGA	(AC)_11_	-	-	-	-	-	33
		R: GTCCTTGCAAGCCATGAA							
ORPM_127	200	F: TCAATGAGGGGTGCCATAAT	(TG)_8_	-	-	-	-	-	33
		R: CTTTCCACTTTTGGCCCTTT							
ORPM_203	209	F: CCACCAGGCATGAGATATGA	(TA)_4_(A/T rich region)	-	-	-	-	-	33
		R: TCAAACCGAAAGGTCAACAA							
ORPM_206	196	F: CCGTGGCCATTGACTCTTTA	(GCT)_7_	-	-	-	-	-	33
		R: GAACCCATTTGGTGCAAGAT							
ORPM_207	199	F: TGCATATTTCACGTGCCTTT	(TC)_8_	+	-	+	-	+	25
		R: CAAAGTGAGGAAGCGTCAGA							
ORPM_349	202	F: GAGCATGAAGCATGAGCAGA	(AC)_16_	-	-	+	-	-	33
		R: TTTTCAGAACCAGGGGAAAA							
PMGC_223	170	F: CGATGAGGTTGAAGAAGTCG	(CTT)_n_	+	+	+	+	+	25
		R: ATATATGTACCGGCACGCCAC							
PMGC_2015	160	F: TTTTGGCATTCAAAGACTTGGC	(GA)_n_	-	-	-	-	-	33
		R: AGTTGATTCCATGTCGTGTCC							
PMGC_2315	143	F: CTGTGGTATTTGTGCAATGTG	(GA)_n_	+	-	+	+	-	33
		R: CAACAGAGCAAACTTGAGTCG							
PMGC_2392	192	F: AAGAGAGATAGCATCACCAAG	(GA)_n_	-	-	-	-	-	33
		R: TATGTCGAGGAAATCCTTAGC							
PMGC_2531	140	F: TAAGAGAATTGGGAGAGCAAC	(GA)_n_	-	-	-	-	-	33
		R: TTTTATCTTTTCCAGTTGTCTAC							
PMGC_2610	114	F: AACACGCAAGAACATACATAAG	(GA)_n_	-	-	-	-	-	33
		R: GATTAACATGTTTCGCTACGC							
PMGC_2647	129	F: CTCGTTAATTAGAGTCGAATTAG	(GA)_n_	-	-	-	-	-	33
		R: TTGTTATCCACTGCCAGTGC							
PMGC_2658	251	F: GCCCTTGAATACCATGAGCG	(GA)_n_	+	-	+	-	+	33
		R: ACCTTCAGTAGATCAGGTTAGTG							
WPMS_7	230	F: ACTAAGGAGAATTGTTGACTAC	(GT)_24_	+	+	-	+	+	33
		R: TATCTGGTTTCCTCTTATGTG							
WPMS_15	193	F: CAACAAACCATCAATGAAGAAGAC	(CCT)_n_	+	+	+	+	+	25
		R: AGAGGGTGTTGGGGGTGACTA							
WPMS_16	145	F: CTCGTACTATTTCCGATGATGACC	(GTC)_n_	+	+	+	+	+	25
		R: AGATTATTAGGTGGGCCAAGGACT							
WPMS_18	245	F: CTTCACATAGGACATAGCAGCATC	(GTG)_13_	-	+	+	-	+	33
		R: CACCAGAGTCATCACCAGTTATTG							

^a^ from The International *Populus* Genome Consortium; n = unknown; F = forward primer, R = reverse primer; + = amplification, - = no amplification.

For *S. eriocephala*, population genetic statistics are summarized in Table 2, Table 3, which shows the number of alleles (A), observed (*Ho*) and expected (*He*) heterozygosities, *p*-values for the Hardy-Weinberg Expected test, and details of each population. Eleven of the 24 markers were successfully amplified in the six populations of *S. eriocephala* (Table 1) and they were single markers. The values of A varied between 1 and 9 (mean 2.95) (Table 2). All 11 markers were polymorphic in at least one population (Table 2). The means of *Ho* and *He* ranged from 0.52 to 0.68 and from 0.43 to 0.56, with mean values of 0.59 and 0.48, respectively (Table 3). These results reveal a new set of polymorphic microsatellite markers in which the genetic diversity within the *S. eriocephala* populations is expressed clearly. 

Tuskan *et al*. [9] using the complete genome of *Populus trichocarpa*, characterized microsatellites markers and predicted that 30–50% of these might transfer to the related *Salix*. Puschenreiter *et al*. [10] successfully amplified 73% of microsatellite markers assayed, 10% of which were polymorphic. Out of 28 *Populus* loci assayed, Lin *et al*. [6] used four loci in their willow study that proved polymorphic (no data were available on the percentage that amplified in willow but were monomorphic). Our amplification success of *Populus* microsatellites was 50% (polymorphic in at least one species), which falls within the range predicted by Tuskan *et al*. [9]; the difference in the percentage of polymorphic amplified loci in the Puschenreiter *et al*. [10] study and ours may stem from the fact that the former used less individuals per species to characterize their loci. In *Salix eriocephala*, Lin *et al*. [6] obtained a higher number of alleles (8–13 *vs.* 1–9) for *Populus*-derived loci, but their population size was much larger than that of populations in our study, which may account for some of the difference. Furthermore, some of our populations were at the extreme northern edge of the species range of *S. eriocephala* and may have lost genetic diversity while migrating northward after glaciations [7]. In all these studies, a majority of markers amplified and were polymorphic for many species, with a number being variably useful depending on the species.

**Table 2:** Results of initial primer screening in six populations of *Salix eriocephala*. Two populations (MON and SHE) are part of a provenance trial maintained at the Montreal Botanical Garden (45°33'41'' N, 73°34'7'' W) and four were collected in nature (BEL, BLA, RAD and VDO). The localization of each population is 49°45.456' N, 77°36.921' W (BEL); 45°41.160' N, 73°51.908' W (BLA); 46°94' N, 70°60' W (MON); 53°41.441' N, 78°06.596' W (RAD); 45°71' N, 64°77' W (SHE) and 48°01.770' N, 77°45.937' W (VDO). Data on markers alleles. The range of allele sizes includes 23 bp of M13 sequence.

Locus	Range of allele sizes (bp)	BEL	BLA	MON	RAD	SHE	VDO	Average
GCPM_1011	207–229	3	4	1	4	1	4	2.83
GCPM_1037	93–188	3	3	5	3	5	5	4.00
ORPM_21	201–229	1	2	3	4	1	2	2.17
ORPM_207	186–221	4	1	1	7	1	4	3.00
ORPM_349	127–133	2	2	2	1	2	2	1.83
PMGC_223	181–219	3	2	2	2	2	5	2.67
PMGC_2315	142–182	3	2	3	2	1	3	2.33
PMGC_2658	205–242	9	6	5	8	4	6	6.33
WPMS_15	161–229	4	2	2	5	1	2	2.67
WPMS_16	137–185	2	3	3	3	3	5	3.17
WPMS_18	245–248	1	1	2	1	2	2	1.50

**Table 3:** Genetic diversity statistics. N = number of individuals sampled in the population.

Population	N	A	*Ho*	*He*	*p*-HWE
BEL	10	3.18	0.60	0.44	0.51
BLA	8	2.45	0.68	0.46	0.15
MON	7	2.82	0.64	0.49	0.40
RAD	12	3.64	0.57	0.43	0.38
SHE	8	2.09	0.54	0.47	0.46
VDO	13	3.55	0.52	0.56	0.15

A = number of alleles; *He* = expected heterozygosity; *Ho* = observed heterozygosity; and *p*-HWE: *p*-values for the Hardy-Weinberg Expected test.

## 3. Experimental Section

**Figure 1:**
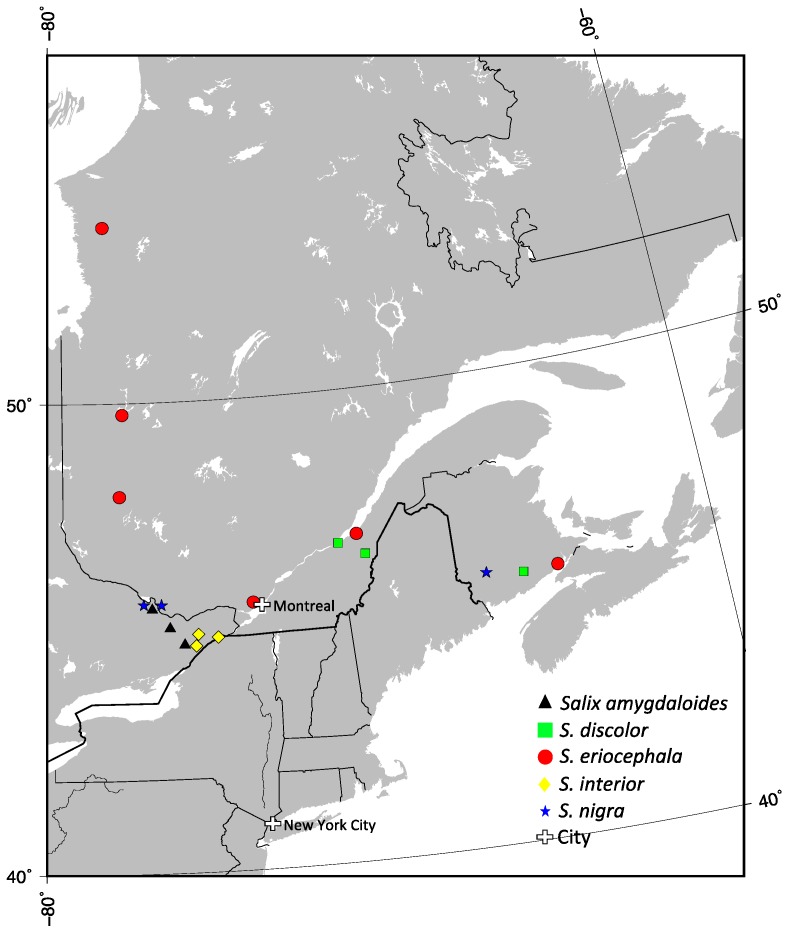
The localization of populations used in this study from Eastern Canada. Triangle for *Salix amygdaloides*, square for *S. discolor*, circle for *S. eriocephala*, lozenge for *S. interior* and star for *S. nigra*.

Six populations of *S. eriocephala* were selected to reflect the wide distribution of this species (Table 1 and Figure 1). These populations were collected at Matagami, QC (BEL, 10 trees); Blainville, QC (BAL, 8 trees); Montmagny, QC (MON, 7 trees); Radisson, QC (RAD, 12 trees); Shepody Creek, NB (SHE, 8 trees) and Val d’Or, QC (VDO, 13 trees). Two are part of a provenance trial maintained at the Montreal Botanical Garden, and four were collected in the wild in 2010–2011. For *S. amygdaloides*, *S. discolor*, *S. interior* and *S. nigra*, we used three populations each from Eastern Canada (Table 1 and Figure 1). The samples of *S. amygdaloides* were collected at Cobden lake, ON (4 trees); Hanlon Marsh, ON (4 trees); and Richmond Fen, ON (4 trees). The samples of *S. discolor* were collected at Levis, QC (10 trees); Montmagny, QC (8 trees); and Norton, NB (10 trees). The samples of *S. interior* were collected at Lafarge Pit, ON (5 trees); Limerick Forest, ON (5 trees); and Long Sault, ON (5 trees). The samples of *S. nigra* were collected at Gagetown, NB (4 trees); Pembroke, ON (4 trees); and Westmeath, ON (6 trees).

Genomic DNA was extracted from 25 mg of leaf material using a modified CTAB method [11]. Leaf tissue was ground for 60 s in a TissueLyser II (QIAGEN) equipped with a 3 mm tungsten ball. 400 μL of 2× CTAB (2% (w/v) hexaldecyltrimethylammonium bromide), 8 μL of mercaptoethanol and 1% (w/v) polyvinylpyrrolidone were added to the powder; the mixture was vortexed and incubated at 65 °C for 90 min. An equal volume of isoamyl alcohol:chloroform (24:1) was then added. Following centrifugation at 4,000 rpm for 30 min at 4 °C, the aqueous phase was collected and the DNA was precipitated by the addition of 2:3 volumes of isopropanol; it was conserved at −20 °C overnight. Then, after centrifugation at 4,000 rpm for 30 min at 4 °C, the pellet was washed with 500 μL of 70% ethanol and centrifuged at 4,000 rpm for 10 min at 4 °C. The DNA pellet was suspended in 100 μL TE (10 mN Tris-HCl pH 8.5, 1 mM EDTA) and stored at −20 °C until analysis.

The International *Populus* Genome Consortium identified 4,200 *Populus* SSRs [6,12,13]. A selection of 24 microsatellite markers (Table 1) was initially assayed to visualize the amplifications. The protocol developed by Schuelke [14] was used to amplify the microsatellite regions. This method fluoresces the PCR products at 700 nm or 800 nm for detection by laser. In this protocol, three primers are necessary: a specific forward primer with a M13 tail at its 5' end, a specific reverse primer, and the fluorescent-labeled M13 primer (5'-AGGGTTTTCCCAGTCACGACGTT-3'). PCRs were carried out in a 10 μL solution containing 0.5 μL of genomic DNA (approximately 50 ng), 0.75× of PCR buffer (BIO BASIC), 0.10 μM of forward primer, 0.25 μM of reverse primer, 0.15 μM of fluorescent-labeled M13 primer (Integrated DNA Technologies), 0.25 mM of dNTPs, 2.25 mM of MgCl_2_, 1 U Taq DNA polymerase (GenScript). An Eppendorf Mastercycler^®^ pro Thermal Cyclers (Eppendorf) under the following cycling parameters: initial denaturation at 94 °C for 3 min followed by 25–33 cycles (Table 1) of 30 s at 94 °C; 30 s at 52 °C, 45 s at 72 °C and followed by a final extension at 72 °C for 5 min was used. The successful amplification was visualized on agarose gel (1%); the results are summarized in Table 1. For *S. eriocephala*, amplification products were separated on a 6.5% KBPlus Gel Matrix and visualized using a 4,300 DNA Analyzer from LI-COR. The SagaGT v3.2 software was used to calculate band sizes. For each locus, we calculated standard population genetic statistics (*Ho*, *He* and *p*-HWE) using GENEPOP v.4.2 [15,16].

## 4. Conclusions

In conclusion, we have added 11 new polymorphic microsatellite markers for *S. eriocephala*. If the loci used by Lin *et al*. [6] are added, a total of 19 loci have been identified and have shown to be polymorphic in this species. The twelve markers characterized in this study are available for further genetic analyses with *S. eriocephala*, *S. amygdaloides*, *S. discolor*, *S. interior* and *S. nigra*. Studies of *Salix* microsatellites increased the molecular tools available for the investigation of willow species and the knowledge of this genus.
